# Equal Effects of Low- and Moderate-Volume Supplementary Plyometric Training on Sprint, Change of Direction Ability, and Lower-Limb Power in Preadolescent Female Handball Players

**DOI:** 10.3390/jfmk9040204

**Published:** 2024-10-25

**Authors:** Konstantinos S. Noutsos, Panagiotis G. Meletakos, Magdalini Kepesidou, Gregory C. Bogdanis

**Affiliations:** School of Physical Education and Sports Science, National and Kapodistrian University of Athens, 17237 Athens, Greece; knutsos@phed.uoa.gr (K.S.N.); meletak@phed.uoa.gr (P.G.M.); magdkepe@phed.uoa.gr (M.K.)

**Keywords:** sprint exercise, team sports, eccentric training

## Abstract

**Background:** This study compared the effects of low- and moderate-volume supplementary plyometric training in preadolescent female handball players. **Methods**: Thirty-nine preadolescent handball players (age: 12.9 ± 0.5 years, maturity offset: −1.50 ± 0.56 years) were randomly assigned into three groups: low-volume plyometric training group (LPT, *n* = 12); moderate-volume plyometric training group (MPT, *n* = 15); and control group (CG, *n* = 12). The supplementary plyometric training program was performed twice per week for 10 weeks, along with normal training (3 times/week). Each supplementary session included two upper- and three lower-body exercises performed for two sets (LPT, 36–54 foot contacts, duration 12 min) or four sets (MPT, 72–108 foot contacts, duration 24 min). The CG performed technical handball drills for 20 min. **Results:** Compared with the control group, both LPT and MPT resulted in similar improvements in modified T-test performance (15.1% and 18.6%, *p* < 0.01, respectively); linear sprint performance over 5, 10, and 20 m (between 5.1% and 8.8%, *p* = 0.02 to 0.001); countermovement jump (28.0% and 22.1%, *p* < 0.05, respectively); and standing long jump (12.6% and 12.5%, *p* = 0.024, respectively). Handgrip strength and overarm shot ball velocity improved similarly in all groups (CG, MPT, and LPT), by 8–11% (*p* < 0.01). **Conclusions:** Very-low-volume supplementary plyometric training performed for 12 min per session for two sessions per week results in considerable improvements in running speed, change of direction ability, and leg power and is as effective as a moderate-volume program. These findings are important for pre-adolescent female team sports players, who may benefit from a minimal but effective supplementary training, resulting in large benefits in leg speed and power.

## 1. Introduction

Handball, as a team sport, requires the development of different physical key abilities such as speed, change of direction ability, leg power, and ball-throwing velocity, which allow for the execution of effective technical–tactical movements in a short time [1]. Plyometric training can play an important role in the development of young female handball players [2]. Previous studies have reported that plyometric training can be safe and effective in improving speed, power, and change of direction ability in children, provided that age-appropriate guidelines are followed [3,4]. In studies involving plyometric jumps, the training volume ranges between 60- and 100-foot contacts per session [5] and may be progressively increased up to around 220-foot contacts per session [6,7]. The determination of the most effective training volume for optimal improvement has been studied mostly in male adults, and there is sparsity of information for young female athletes and specific popular sports such as handball [5]. Previous randomized controlled studies in very young male soccer players (8–13 years old) have shown that prepubertal boys can considerably improve leg speed, strength, power, and change of direction ability [8,9], but information on young female athletes is limited to ages between 13 and 16 years [10,11].

Training volume is an important variable in plyometric jumps training. This is because plyometric exercises pose high loads on the musculoskeletal structures, which are susceptible during adolescence and especially in females who have different anatomical characteristics and strength compared with males [3,12,13]. Thus, the determination of the lowest effective training volume for pre-adolescent female athletes has implications not only for their performance, but also for their musculoskeletal health. Previous research [7] examined the effect of low- (50- to 120-foot contacts per session) and high-volume plyometric training (110- to 220-foot contacts per session, 2 sessions/week for 8 weeks) on physical fitness measurements in preadolescent male soccer players (U-13). The results showed similar performance improvement in sprint-time, change-in-direction, and jumping ability between the two programs. Ramirez-Campillo et al. [14] studied the effect of plyometric training volume in 17-year-old high-school boys and concluded that a high training volume was necessary to improve explosive performance (such as speed), but a moderate plyometric volume was adequate when the training surface was hard.

In the sport of handball, athletes are required to have explosive strength and speed in both the lower and upper limbs. However, there are only few studies examining the effects of plyometric training in young female handball athletes. Hammami et al. [10] applied a lower- and upper-body plyometric program with a relatively low number of contacts (60–90) in 16-year-old female handball players and found increases in sprint speed, change of direction, jumping ability, and repeated change of direction. Similar findings have been reported by Chaabene et al. [15] for 16-year-old female handball players, who substituted part of training (handball-specific drills) with plyometric training (90–120-foot contacts). The effects of plyometric training using both the lower and upper limbs in younger (13-year-old) female handball athletes has only been examined in one study [11], which reported significant improvements in sprint, change of direction, vertical and horizontal jumping, upper-limb strength, and balance.

Taking all the available information into consideration, it is evident that there is a need to examine whether a very low-volume plyometric training program (<50-foot contacts) requiring only a short time to be performed, would be effective and safe in pre-adolescent female team sports athletes, who may benefit from that in terms of both performance and health. Hence, the aim of the current study was to examine if low- (36–54-foot contacts) and moderate-volume plyometric training (72- to 108-foot contacts) would improve lower- and upper-limb power, in an attempt to define the lowest volume of supplementary plyometric training that is effective. For this purpose, a randomized controlled trial was conducted over 10 weeks. Our hypothesis was that both methods of plyometric training would equally enhance sprint, change-in-direction ability, and lower-limb power in preadolescent female handball players.

## 2. Materials and Methods

### 2.1. Participants

The sample of the present study consisted of thirty-nine female preadolescent handball players, aged between 11 and 13 years. The subjects were members of academies practicing systematically in the sport of handball to develop technical and tactical skills and to improve their physical condition. The study was conducted during the 2023–2024 pre-season from September to November. For inclusion in the study, the participants had to have at least one year of handball training experience, while they were excluded if they had a recent injury. Parents or guardians were informed that they were free to discontinue their child’s participation in the study at any stage they wished, without providing any explanation. After accepting their children’s participation, the parents or guardians signed the consent form. The procedure of the study was approved by the Ethical Committee of the School of Physical Education and Sports Science of the National and Kapodistrian University of Athens (Approval number: 1515/19-04-2023). Baseline physical characteristics are presented by group in Table 1.

### 2.2. Experimental Design

The participants in a single-blinded randomized controlled design were randomly assigned into one of the following three groups: the low-volume plyometric training group (LPT, *n* = 12), the moderate-volume plyometric training group (MPT, *n* = 15), and the control group (CG, *n* = 12). Players were members of three teams, whose coaches agreed to collaborate with our research group and follow the same overall training program. Thus, the random assignment was carried out at a team level, and the only difference in training schedules between teams was the supplementary program. To ensure proper and consistent technical execution of the plyometric training prior to the start of the intervention period LPT and MPT, four familiarization sessions were performed over two weeks. Verbal feedback was given during each session, which aimed to minimize contact time and increase flight time where possible. In the second week of adaptation, all female athletes of the study underwent their first measurements.

The supplementary plyometric training program was performed twice per week for a total of 20 sessions, with at least 48 h between sessions. Three lower-body and two upper-body exercises were performed in each session for two (LPT) or four sets (MPT) per exercise. For the LPT group, the duration of the supplementary plyometric training was approximately 12 min, while for the MPT group, it was approximately 24 min. The only difference between the two experimental groups was the training volume (number of sets performed), while the exercises performed were the same. The CG performed technical handball drills for 20 min, at the same part of the main session.

**Figure 1:**
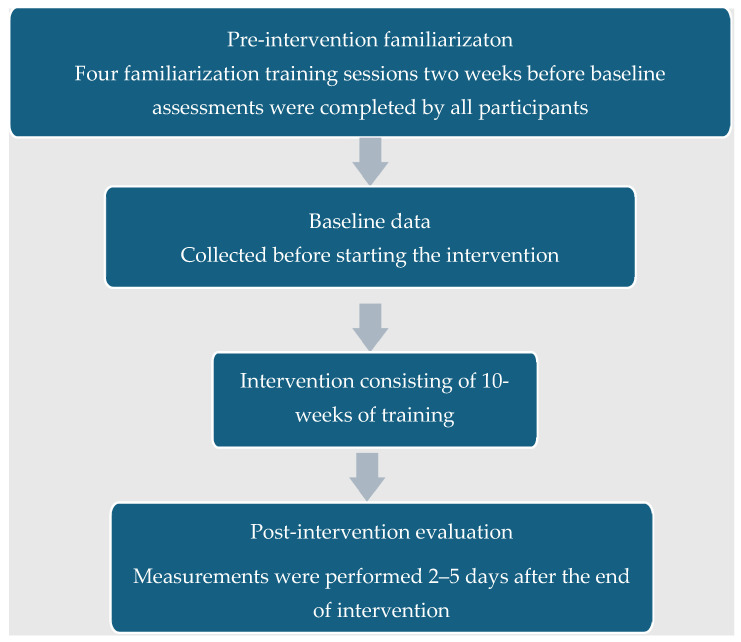
Timeline of measurements and intervention.

Before and after training, anthropometric measurements, a modified agility T-test, and linear sprint measurements were performed in one session, while vertical jump, standing long jump, maximal handgrip strength, and ball-throwing velocity were measured in a second session, at least 48 h apart. The same motor performance measurements were performed after ten weeks of training. Fitness tests were preceded by a standardized warm-up which included 5 min of low-intensity running, coordination exercises, and running drills, followed by static and dynamic stretching, lasting 15 min. Participants were asked not to engage in vigorous physical activity the day before or on the day of testing. All tests were performed in the same order, with full recovery (6–8 min) between tests (Figure 1). 

### 2.3. Measurements

#### 2.3.1. Anthropometric Measurements

Body height was measured with a stadiometer (Seca model 220, SECA, Hamburg, Germany) to the nearest 0.1 cm, and body mass was recorded using a portable scale (Seca 770; Seca, Hamburg, Germany) to the nearest 0.1 kg. Sitting height was measured as the distance from the highest point on the head to the base-sitting surface to the nearest 0.1 cm. Body mass index (BMI) was calculated from body mass and body height. Body fat percentage (BF%) was estimated from four skinfold measurements (biceps and triceps, suprailiac and subscapular [16].) Maturity offset was calculated from anthropometric measurements (height, sitting height and chronological age) using the modified maturity offset equation for girls [17]. Maturity offset = −9.376 + (0.0001882 × leg length × sitting height) + (0.0022 × age × leg length) + (0.005841× age × sitting height) − (0.002658 × age × weight) + (0.07693 × weight by height ratio).

#### 2.3.2. Modified Agility T-Test (MAT)

To measure change-in-direction ability, the modified agility T-test was selected. The time to complete the test as fast as possible was recorded using electronic photocells placed at the start line of the test, which coincided with the finish line. The participants accelerated in a straight line for 5 m and touched the base of the first cone with their hand. Then, they moved sideways until they touched the base of the cone with the hand placed 2.5 m to the left, reversed their sideways movement until they touched the right cone with their hand, and again moved sideways to the left until they touched the middle cone with their hand. From there, they ran backwards until the starting point. Participants performed three trials, and the best time was kept for analysis to the nearest 0.01 s. The test result was not considered valid if a participant crossed one leg in front of the other or failed to touch the base of the cone [18]. A 5 min rest period was allowed between each test. Reliability was high, as assessed by the intraclass correlation coefficient (ICC = 0.874).

#### 2.3.3. Linear Sprint

The 5 m, 10 m, and 20 m linear sprint time (T5, T10, and T20, respectively) was recorded during a maximal 20 m sprint using four pairs of photocells (FitLight Sports Corp, Aurora, ON, Canada) placed 0.75 m above the ground to ensure that they captured trunk movement. The test started from a standing position 30 cm behind the first pair of photocells, and the participants sprinted as fast as possible. Two trials were performed with at least 3 min of rest in between. The fastest time was recorded for further analysis with an accuracy of 0.01 s. Reliability for 5, 10, and 20 m performance was high (ICC = 0.870–0.916).

#### 2.3.4. Countermovement Jump (CMJ)

The athletes started from a standing position, with both feet–shoulder width apart, head in a straight line, with knees fully extended, and hands akimbo. The jump was preceded by a reverse downward movement by bending the knees to an angle of approximately 90 degrees, which was visually checked by an examiner. The athlete was instructed to jump as high as possible, and verbal encouragement was given before each trial. Participants were instructed to maximize jump height and minimize ground contact time [19]. Three attempts were performed with 1 min rest in between, and the best was used for further processing. Jump height (cm) was calculated from flight time using the “My Jump 2” app running on an iPhone (iPhone 12 mini 64 GB; Apple, Cupertino, CA, USA) at a sampling rate of 240 Hz and resolution of 2340 × 1080 pixels. The validity and reliability of this method of measurement has been previously established [20]. In the present study, the ICC for the CMJ was 0.943. 

#### 2.3.5. Standing Long Jump (SLJ)

The participants were standing with their feet–shoulder width apart behind a starting line and their arms hanging loosely at their sides. They then jumped as far as possible using a countermovement, and the distance of the jump was measured in centimeters from the starting line to the heel of the foot closest to that line. Athletes were instructed to land on both feet simultaneously and were not allowed to fall forward or backward. Two attempts were made, and the longest jump was kept for further analysis. Reliability of SLJ performance was high (ICC = 0.949).

#### 2.3.6. Hand Grip Strength (HGS)

The assessment of the maximum isometric grip strength was carried out from a sitting position, with the elbow of the examined arm at an angle of 90 degrees [21] and the wrist in a neutral position. The dynamometer handle (Jamar Plus, Performance Health, Warrenville, IL, USA) was adjusted to fit each athlete’s hand. Three 5 s maximum isometric contractions of each arm were performed with a 1 min rest interval, and the best trial for each hand was recorded in Kg. Reliability of HGS measurement was high (ICC = 0.912)

#### 2.3.7. Ball Velocity (BV)

The maximum velocity of the ball during a penalty throw from 7 m was recorded. Three attempts were performed, with 45 s of rest in between, and the best performance will be used for further analysis [22]. Peak ball velocity was recorded using a high-performance sports radar (Stalker Pro 2 Radar Gun, Applied Concepts, Inc./Stalker Radar, Richardson, TX, USA) positioned behind the goal pointing at the shooting arm, and speed was measured in kilometers per hour (km/h). Reliability of BV measurement was high (ICC = 0.919)

### 2.4. Plyometric Training Programs

**Figure 2:**
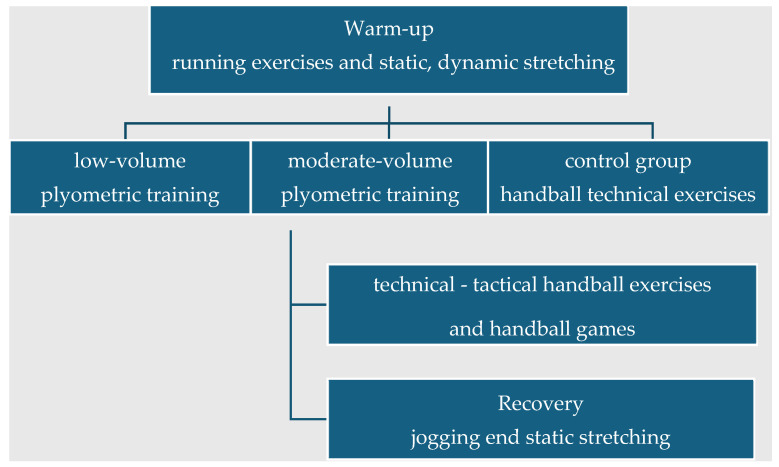
Training session structure on the days of plyometric training.

The normal training of the athletes included three 80–90 min sessions per week. Supplementary plyometric training was performed twice per week for 10 weeks (a total of 20 sessions). At least 48 h separated the plyometric training to allow for full recovery [23]. Each training session started with a 10 min warm-up, which included running drills, followed by 15 min of static and dynamic stretching. After the common warm-up, the LPT and the MPT groups performed supplementary plyometric training while the CG performed technical handball drills for 20 min. For the LPT group, the duration of the supplementary plyometric training was approximately 12 min, while for the MPT group it was approximately 24 min. After performing the supplementary plyometric training or the control (handball drills), the athletes continued with handball training, which included technical and tactical elements and handball games for approximately 30 min. Each training session ended with light running and static stretching for 7–10 min (Figure 2).

The training program followed by all groups was controlled by the experimenters for the entire duration of training (10 weeks) and was similar in all groups regarding the warm-up, cool-down, and all technical–tactical elements and games. Three lower-body and two upper-body exercises were performed in each session for two (LPT) or four sets (MPT), as detailed in Table 2. The only difference between the two experimental groups was the training volume (number of sets performed), while the exercises performed were the same. The recovery interval between all exercises was 1:10 (exercise: rest ratio), while there was a progression of exercises and total training volume every two weeks to ensure continuing improvements [24] (Table 2).

### 2.5. Statistical Analysis

Statistical analysis was conducted by using SPSS Statistics Ver. 23 (IBM Corporation, Armonk, NY, USA). A Kolmogorov–Smirnov test showed that all dependent variables did not deviate from the normal distribution, making feasible the application of parametric procedures. Repeated-measures multivariate analysis of variance (RM-MANOVA), followed by mixed-repeated-measures analysis of variance (ANOVA), were used to compare the means for the three groups at the pre-measurement stage (i.e., the demographic and anthropometric characteristics such as the age, maturity offset, height, weight, and BMI) and also the eight performance tests at baseline and following training. The effects of training were compared with this repeated-measures approach. Also, the change in performance, i.e., the difference between the pre- and the post-training test measurements, was calculated and subsequently analyzed using a multivariate analysis of variance, followed by univariate ANOVA. This examined if the changes between the pre- and post-measurements varied significantly between the three groups, while post hoc pairwise comparisons with Bonferroni correction located the differences. Effect sizes were calculated using Cohen’s d, which was classified as small (d = 0.2), medium (d = 0.5), and large (d ≥ 0.8). The level of significance was set at 0.05.

### 2.6. Sample Size Calculation

The test chosen for the power analysis was the 10 m linear sprint time (T10) and, specifically, the difference in the reduction in the sprint time between the control group and any of the two experimental groups. According to previous studies, this difference was expected to be about 0.13 s, with a standard deviation in each group of about 0.1 s. According to the calculations, if these differences were repeated, then we would need to measure 10 experimental subjects and 10 control subjects to be able to reject the null hypothesis that the population means of the experimental and control groups are equal with the probability (power) of 0.80. The Type I error probability associated with this null hypothesis test was set at 0.05. Calculations were performed with the software PS Power and Sample Size Calculations Version 3.1.6, 2018 [25].

## 3. Results

The repeated-measures multivariate analysis of variance showed that the three groups were equivalent with regard to the baseline characteristics (Pillai’s Trace = 0.265, F (6,70) = 1.785, *p* = 0.115). Subsequent univariate analyses showed that there were no between-group differences in age (*p* = 0.697), maturity offset (*p* = 0.189), and height (*p* = 0.051), but the control group had higher body mass (*p* < 0.01) and BMI (*p* < 0.01) than the two experimental groups (Table 1).

The repeated-measures multivariate analysis of variance showed that the three groups were equivalent with regard to the eight pre-training measurement tests (Pillai’s Trace = 0.396, F (16,60) =0.926, *p* = 0.545). Subsequent univariate analyses proved that this equality was true for all pre-measurement tests of MAT (*p* = 0.611), T5 (*p* = 0.053), T10 (*p* = 0.265), T20 (*p* = 0.651), CMJ (*p* = 0.327), SLJ (*p* = 0.906), HGS (*p* = 0.912), and BV (*p* = 0.367) (Table 3).

After establishing the equality of the three groups with regard to the baseline characteristics and the pre-training performance tests, the repeated ANOVAs showed a group-by-time interaction for MAT, T5, T10, T20, CMJ, and SLJ (*p* < 0.001) but only a time effect for HGS and BV, suggesting that all groups equally increased HGS and BV overtime. Subsequent post hoc tests revealed that there was no change in T5, T10, T20, and SLJ for the control group. However, there was a small improvement in MAT and CMJ for the control group. 

**Figure 3:**
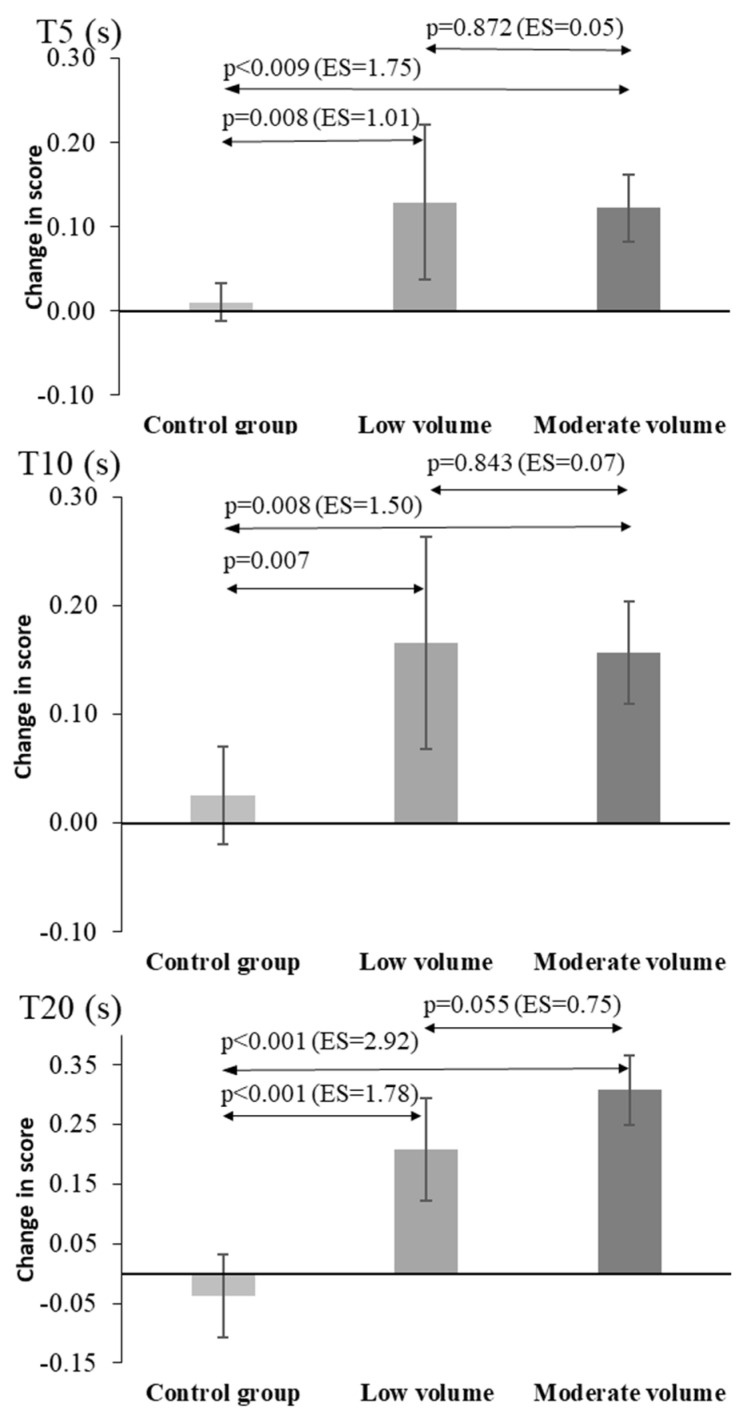
Mean values and 95% confidence intervals (CI) of the performance improvement (difference between the pre- and post-training test results) for the three groups. The pairwise comparisons between the three groups are indicated with arrows, with the corresponding *p* values and effect sizes in parentheses. T5, T10, and T20: performance times during 5, 10, and 20 m sprinting.

**Figure 4:**
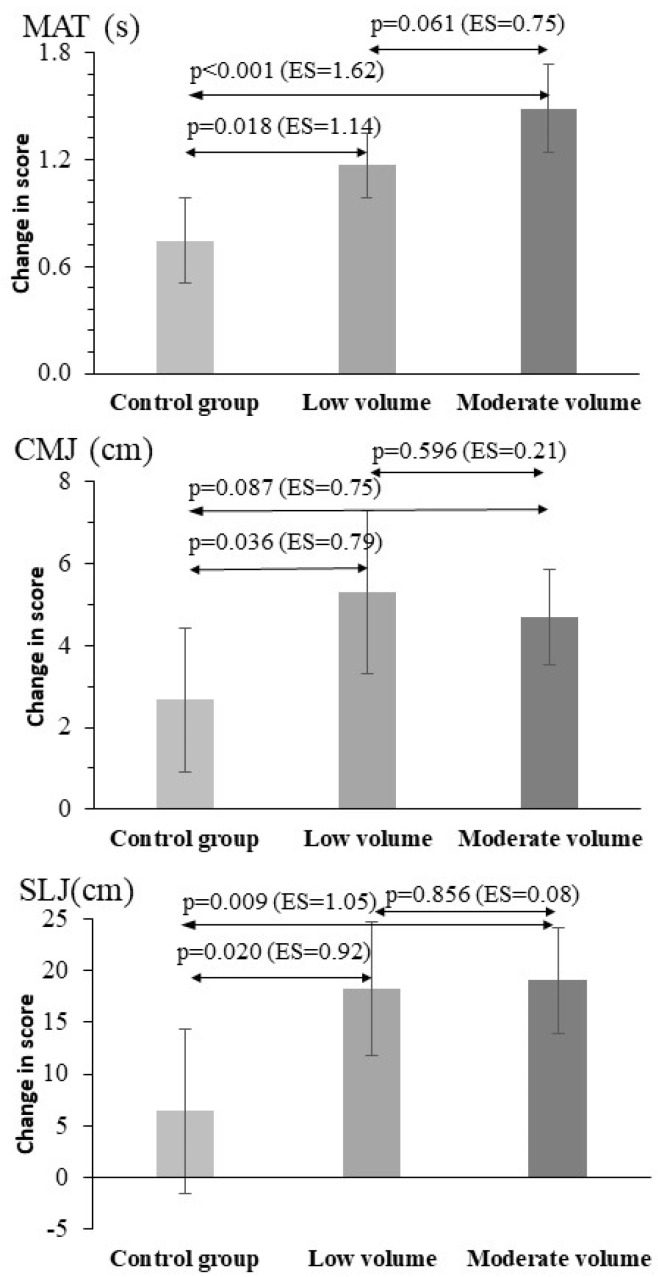
Mean values and 95% confidence intervals (CIs) of the performance improvement (difference between the pre- and post-training test results) for the three groups. The pairwise comparisons between the three groups are indicated with arrows, with the corresponding *p* values and effect sizes in parentheses. MAT: modified agility T-test; CMJ: countermovement jump performance; SLJ: standing long jump performance.

**Figure 5:**
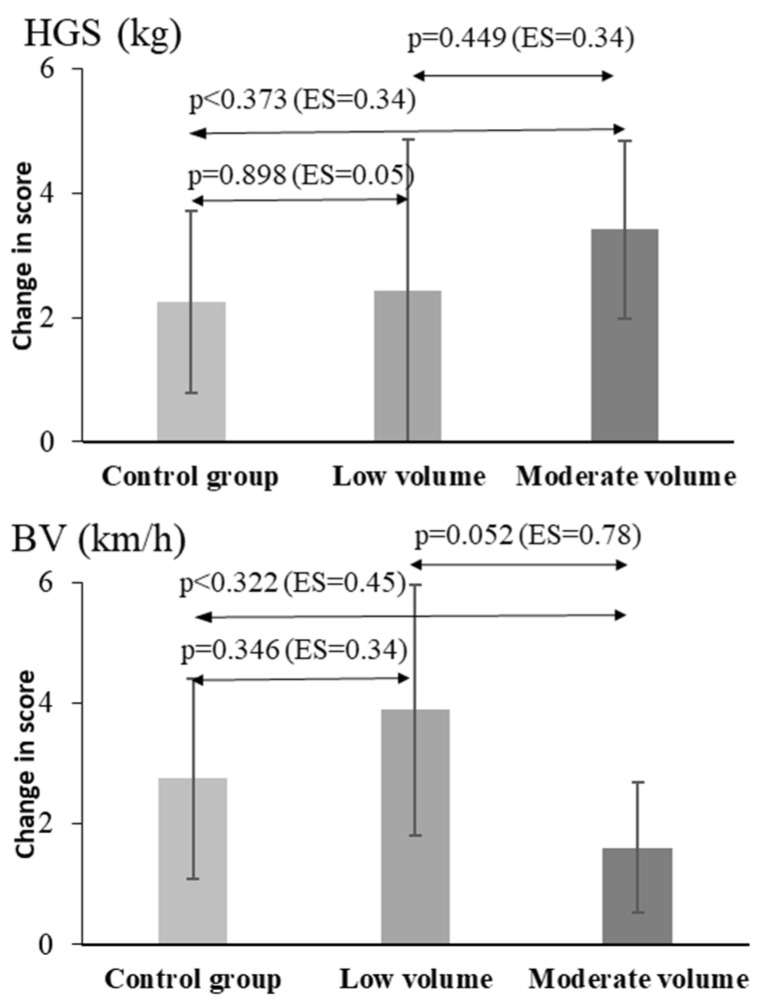
Mean values and 95% confidence intervals (CI) of the performance improvement (difference between the pre- and post-training test results) for the three groups. The pairwise comparisons between the three groups are indicated with arrows, with the corresponding *p* values and effect sizes in parentheses. HGS: handgrip strength; BV: ball velocity during a penalty throw.

The calculated differences between the pre- and post-measurements reflected the above findings (Figure 3, Figure 4 and Figure 5). When comparing the changes in performance between low- and moderate-volume supplementary training for MAT, T5, T10, T20, CMJ, and SLJ, two main conclusions can be drawn. Both interventions (low- and moderate-volume training) caused a large improvement compared to control. The post hoc pairwise comparisons showed significant improvements for the low- and moderate-volume plyometric training groups compared with the control group for all sprint times (between 5.1% and 8.8% for T5, T10, T20, *p* = 0.02 to 0.001, Figure 3), for MAT (15.1% and 18.6%, *p* < 0.01, respectively, Figure 4), for CMJ (28.0% and 22.1%, *p* < 0.05, respectively), and for SLJ (12.6% and 12.5%, *p* = 0.024, respectively, Figure 4). Notably, the control group also showed improvements compared with the baseline values in MAT (*p* < 0.01), CMJ (*p* < 0.01), HGS (*p* < 0.01), and BV (*p* < 0.01) tests but not in sprint performance or SLJ (*p* > 0.69).

The improvements in performance in the six tests were similar between the two experimental groups, thus proving the primary working hypothesis of the study. Although not statistically significant (*p* > 0.05), the effect size of the comparison between the low- and moderate-volume groups was moderate (0.75) for T20 and MAT, suggesting a possibly greater effect of moderate volume training, while the opposite was observed based on effect sizes for BV, where the low-volume group had superior improvement (ES = 0.78, Figure 5).

## 4. Discussion

This study showed that ten weeks of supplementary plyometric training performed twice per week in pre-adolescent female athletes resulted in considerable improvements in speed, change-in-direction ability and jumping performance, while it did not augment gains in handgrip strength and ball-throwing velocity, which equally improved in all groups. Importantly, the present study demonstrated that very-low-volume (only 12 min per session) supplementary plyometric training per session is adequate to bring about the similar improvements in performance as higher-volume plyometric training (24 min per session). This finding has large practical significance, especially because the participants were pre-adolescent female team sports athletes, who may train safely and effectively with very-low-volume plyometric training during this developmental stage.

The effectiveness of a very-low-volume supplementary plyometric training for young female athletes is an important finding of the present study. The fact that there was minimal or no improvement in the CG indicates that a supplementary plyometric training is necessary for these young female team sports players, as previously shown for individual sports athletes [26]. On the other hand, the results showed that when the supplementary training volume was doubled, there was no further improvement in performance, confirming the observations of De Villarreal et al. [27], who argued there may be an upper threshold in training volume beyond which there is no additional benefit.

A larger-percentage improvement in performance indicators in both groups (low- and moderate-volume group) was observed in CMJ (22–28%), while sprint performance improved much less (5.1–7.5%). Interestingly, change-in-direction ability, which is a combination of speed and leg power, improved 15–19%, demonstrating the importance of leg power for movements requiring acceleration, deceleration, and changes in direction. Athletes with a well-developed change-in-direction ability have a technical and tactical advantage over their opponent as they may have an increased opportunity to outrun their opponent and move into space during attacks [28]. The results of our study showed significant improvements in change-in-direction ability performance for the LPG and MPG groups compared to the CG. These improvements in change-in-direction ability are consistent with previous studies. Hammami et al. [10] observed a significant improvement in change-in-direction ability (T-test: *p* < 0.01, Δ% = 14.5, d = 0.993) after 9 weeks of plyometric training in U14 female handball athletes. Also, Chaabene et al. [15] found an improvement in change-in-direction ability [T-test time (ES = 1.46)] in young female handball players after an 8-week plyometric training program. Change-in-direction ability is mainly manifested through the rapid change of direction, achieved by acceleration and deceleration of the lower limbs, in response to various situations or stimuli [29]. Much of the plyometric training applied incorporated vertical, horizontal, and unilateral jumps that likely increased MAT performance.

In the post-intervention assessments of linear sprint time, significant improvements in the LPT and MPT groups were observed compared to the CG. The data of our study confirm findings of other studies which also performed plyometric training in female handball athletes and achieved improvements in sprinting at different distances. Hammami et al. [10] found significant improvements in 20 m performance (*p* = 0.02, d = 0.557) after 9 weeks of combined upper- and lower-limb plyometric training in U14 female handball athletes. More recently, another study [11] of young female handball athletes (15.8 ± 0.2 years) conducted over 10 weeks showed improvements in 5 m linear speed (*p* = 0.001, d = 0.82), 10 m (*p* = 0.002, d = 2.37), and 20 m (*p* = 0.001, d = 1.19). Improvements in linear sprinting ability with plyometric training can be largely attributed to neurological adaptations, including improved motor unit activation, enhanced muscle coordination, and refined reflex control [30]. Also, the greatest benefits of plyometric training on sprint performance depend on the speed of muscle action, stretch–shortening cycle (<0.25 s), used in training [31]. The plyometric training in our study included horizontal jumps, and, according to Ramirez-Campillo et al. [4], the direction of muscle force application may contribute to the magnitude of the observed improvements. Therefore, performing horizontal jumping exercises may have contributed to sprint improvements, given the importance of horizontal force production in sprinting [32,33].

Executing a vertical jump is an important ability in handball players in their attempt to overcome the opponent’s block and direct the ball into the goal. In the present study, the results showed significant improvements in CMJ by the LPT and MPT groups compared to the CG. Similar results were observed by researchers in other studies examining the effect of plyometric training on CMJ. Hammami et al. [10] also found large improvements (d = 1.17) in CMJ performance in young female handball players after a 10-week compound training program. Similarly, Chaabene et al. [15] observed significant increases in vertical jump performance (ES = 0.57) following plyometric training in young female handball athletes. In their research, Iacono et al. [34] confirmed that vertical plyometric training was more effective for vertical jumps. The main mechanism explaining the effects of plyometric training may be an improvement of stretch–shortening cycle performance which is involved with most movement patterns in competitive sports [4]. The improvement in jump performance after plyometric training can be partially attributed to positive neuromuscular adaptations to high eccentric forces and corresponding improvements in vertical jump ability [27,30,35,36]. Thus, improving the CMJ through training methods such as plyometric training using vertical jumps may be of great importance to handball training. In addition, SLJ showed significant, but smaller, improvements for both the LPT and MPT groups compared to the CMJ. Gaamouri et al. [2] noted that the plyometric training program caused a significant increase in SLJ values in young female handball athletes aged 15.8 ± 0.2 years. Arazi et al. [37] also observed similar improvements in long jump [8.3 (ES = 0.3) vs. 12.7 (ES = 0.57) cm]. A recent meta-analysis by Rocha et al. [38] concluded that horizontal jumps are more effective in enhancing horizontal performance. However, the smaller improvement in SLJ compared with CMJ may be due to the higher technical demands of SLJ, which involves effective push-off to an optimum angle and an effective landing technique [39].

A consistent level of grip strength is necessary for handball players to increase their control and efficiency during defense (direct physical contact between opposing players and performing various movements with the ball [40]). Handgrip strength testing is often performed to assess upper-body strength in game sport athletes is the handgrip strength [41]. However, isometric handgrip strength depends on age, gender, training, and body dimensions [42], while it may not be considered as a specific strength test for handball. The present results showed no additional improvement in HGS for the LPT and MPT groups compared to the CG, which was expected due to the focus of plyometric training on other muscle groups, i.e., lack of training specificity. In the study by Hammami et al. [11], an upper- and lower-body plyometric training program resulted in increases in handgrip strength after 9 weeks of plyometric training in female handball athletes under 14 years of age. In contrast with the present study, the program used by these researchers included more upper-body exercises, and this explains the different findings.

A successful throw or shot during a match depends both on speed [43] and accuracy [44]. The findings of our study did not reveal greater improvement of BV in the LPT and MPT groups compared to the CG. Chelly et al. [45] noted increased throwing speed (19% penalty throw) after 8 weeks of plyometric training (plyometric push-ups). In the studies by Dahl and Van Den Tillaar [46] and Soto Garcia, et al. [47], who performed a plyometric training protocol for eight weeks, it was observed that the ball-throwing speed did not improve. Throwing speed in handball is highly dependent on throwing technique and upper- and lower-limb strength [48]. A possible explanation for the equal improvement of ball velocity during the penalty throws in all groups, including the CG, is the lack training specificity, since the supplementary plyometric training included push-ups and medicine ball throws, which are quite dissimilar to a penalty throw both in terms of technique and movement specificity. Therefore, future studies should examine these aspects.

The comparison between the two groups after the intervention showed statistically non-significant differences for all parameters. This finding suggests that a short-term plyometric training session lasting approximately 12 min (LPT) is an equally effective and more time-efficient volume stimulus compared to a double volume training (MPT) because they both induce similar improvements in change of direction ability, speed, jumping ability, maximal handgrip strength, and ball-throwing velocity.

The following limitations should be considered when interpreting the current results: First, this study is limited to preadolescent female handball players, and therefore, the results cannot be generalized to the entire population. Second, the intervention training was carried out at the same time as the handball training, which may interact with the effects of this supplementary training, affecting the final performance results. This was partially accounted for by including a control group. Further research should examine the application of plyometric training to larger samples of female players of a wider age-range, using both lower and higher volumes of supplementary training.

## 5. Conclusions

Very-low-volume plyometric training performed for 12 min per session for two sessions per week results in considerable improvements in running speed, change-in-direction ability, and leg power and is as effective as a moderate-volume program of twice as high duration (24 min per session). These findings are important for pre-adolescent female team sports players, who may benefit from a minimal volume, but effective, supplementary training, inducing relatively large benefits in leg speed and power. In practical terms, it may be suggested that coaches can replace a small portion of standard handball training with a 12 min plyometric training program to enhance the performance of female handball players in ten weeks. This information may have important practical implications in training strategies to optimally design supplementary programs combining low load and high effectiveness.

## Figures and Tables

**Table 1:** Baseline characteristics of the three groups. Means and standard deviations.

Characteristic	Control Group (*n* = 12)		Low Volume Group (*n* = 12)		Moderate Volume Group (*n* = 15)	
	Mean	SD	Mean	SD	Mean	SD
Age (y)	12.8	0.5	12.9	0.5	12.9	0.6
Maturity offset (y)	−1.27	0.36	−1.67	0.74	−1.56	0.51
Body mass (kg)	64.9 *	6.0	52.0	8.0	56.7	7.2
Height (cm)	162.8	3.6	157.6	5.2	161.7	6.3
Sitting height (cm)	85.0	2.6	80.6	5.1	81.9	3.6
BMI (Kg/m^2^)	24.5 *	2.6	20.9	3.0	21.7	2.3
Body fat (%)	27.2 *	1.7	24.5	3.2	24.3	2.3
Body fat (kg)	17.7 *	2.3	12.9	3.4	13.8	2.5
Lean mass (kg)	47.1 *	3.9	39	4.7	42.7	4.9

*: *p* < 0.01 from low- and moderate-volume groups.

**Table 2:** Description of the plyometric training program for the low- (LPT) and moderate-volume groups (MPT).

			LPT	MPT
Week	Body Part	Plyometric Training	Set × Reps (Duration)	Set × Reps
1–2	Lower body	Forward and Backward Hurdle Hops, (hurdle height: 15 cm)	2 × 6 (4 s)	4 × 6
		Lateral Hurdle Jumps, (hurdle height: 15 cm)	2 × 6 (4 s)	4 × 6
		Vertical Jumps in Place	2 × 6 (9 s)	4 × 6
		Foot contacts	36	72
	Upper body	Plyometric Pushups	2 × 4 (8 s)	4 × 4
		Medicine ball (1 kg) Two-Hand Overhead Throw	2 × 4 (7 s)	4 × 4
		Duration	11.7 min	23.5 min
3–4	Lower body	Right leg. Single-leg Hurdle Hops, (hurdle height: 15 cm)	2 × 7 (4 s)	4 × 7
		Left leg. Single-leg Hurdle Hops (hurdle height: 15 cm)	2 × 7 (4 s)	4 × 7
		Standing Vertical Jump	2 × 6(8 s)	4 × 6
		Foot contacts	40	80
	Upper body	Plyometric Pushups	2 × 4 (8 s)	4 × 4
		Medicine ball (1 kg) Backwards Throw	2 × 4 (9 s)	4 × 4
		Duration	12.1 min	24.2 min
5–6	Lower body	Hurdle Hops	2 × 7(6 s)	4 × 7
		Zig-zag Jump	2 × 7(8 s)	4 × 7
		Standing Triple Jump	2 × 3 (6 s)	4 × 3
		Foot contacts	46	92
	Upper body	Medicine ball (1 kg) Chest Pass	2 × 4 (7 s)	4 × 4
		Medicine ball (1 kg) Rainbow Slams	2 × 4 (6 s)	4 × 4
		Duration	12.1 min	24.2 min
7–8	Lower body	Single-leg (right) Zig-Zag Hops	2 × 7 (7 s)	4 × 7
		Single-leg (left) Zig-Zag Hops	2 × 7 (8 s)	4 × 7
		Standing Triple Jump	2 × 3 (6 s)	4 × 3
		Foot contacts	46	92
	Upper body	Medicine ball (1 kg) Squat Throw	2 × 4 (7 s)	4 × 4
		Medicine ball (1 kg) Rainbow Slams	2 × 4 (6 s)	4 × 4
		Duration	12.5 min	25.0 min
9–10	Lower body	Forward and Backward Hurdle Hops, (hurdle height: 15 cm)	2 × 9 (7 s)	4 × 9
		Lateral Hurdle Jumps, (hurdle height: 15 cm)	2 × 9 (7 s)	4 × 9
		Alternate Leg Bounds	2 × 9 (7 s)	4 × 9
		Foot contacts	54	108
	Upper body	Medicine ball (1 kg) Single-Arm Throw	2 × 4 (6 s)	4 × 4
		Medicine ball (1 kg) Side Throw	2 × 4 (6 s)	4 × 4
		Duration	12.1 min	24.2 min

**Table 3:** Pre-training measurements for the three groups. Means and standard deviations.

	Control Group		Low-Volume Group		Moderate-Volume Group	
Test	Mean	SD	Mean	SD	Mean	SD
MAT (s)	7.87	0.62	7.7	0.48	7.9	0.52
T5 (s)	1.48	0.11	1.43	0.14	1.37	0.06
T10 (s)	2.44	0.15	2.39	0.16	2.35	0.11
T20 (s)	4.11	0.27	4.07	0.22	4.07	0.15
CMJ (cm)	19.54	3.69	21.69	5.21	21.58	2.88
SLJ (cm)	154.25	18.09	154.88	24.09	157.31	14.76
HGS (kg)	27.02	3.69	27.06	6.71	27.68	2.73
BV (km/h)	34.58	4.25	34.50	5.16	36.67	4.12

MAT: modified agility T-test; T5: 5 m linear sprint time; T10: 10 m linear sprint time; T20: 20 m linear sprint time; CMJ: counter movement jump; SLJ: standing long jump; HGS: handgrip strength; BV: ball velocity.

## Data Availability

Data will be provided upon reasonable request from the first author.
